# Child Homicide: A Global Public Health Concern

**DOI:** 10.1371/journal.pmed.1002004

**Published:** 2016-04-26

**Authors:** Delan Devakumar, David Osrin

**Affiliations:** 1 Institute of Epidemiology and Health Care, University College London, London, United Kingdom; 2 Institute for Global Health, University College London, London, United Kingdom

## Abstract

In this perspective, Delan Devakumar and David Osrin discuss Abrahams and colleagues’ findings in the context of evidence about child homicide in different countries, and consider etiology along with implications for child protection and prevention.

Despite recent increases in child survival, some 5.9 million children still die each year, and reducing global childhood mortality remains a public health priority [1]. The greatest numbers of deaths are due to infections, intrapartum events, and preterm births, but reductions in all causes of mortality are needed to reach the Sustainable Development Goal target of 25 deaths for every 1,000 live-born infants. In a research article in *PLOS Medicine*, Naeemah Abrahams and colleagues shed some light on the occurrence of child homicide [2]. Tragedy radiates from such events, backward in time to the pressure and emotional burden that might lead people, willfully or not, to end the life of a child, and forward to the effects on parents and families. Children who survive attempted homicide may go on to suffer long-term traumatic consequences [3].

Abrahams and colleagues examined data on homicides of children aged under five years in South Africa. They began by assessing records of unnatural deaths over a single year, 2009, in postmortem reports from a sample of medico-legal laboratories of different sizes and in urban and rural settings. They contacted investigating police officers and interviewed them to gather more information. Most of the deaths (74.4%) were of infants less than one year old (of whom 53.2% were neonates in their first 28 days of life). Mortality rates among neonates, infants, and children aged 1–4 years were 19.6, 28.4, and 1.0 per 100,000 live births, respectively. These figures are substantial: assuming an overall neonatal mortality rate of 12–14 per 1,000, the neonatal mortality estimate from homicide in the present study corresponds to ~1.5% of all neonatal deaths in South Africa in 2009.

Were the high rates of child homicide found in the study a product of South Africa’s high aggregate homicide rate (31 per 100,000), or were they unexpected [4]? Global homicide rates average 6.2 per 100,000, but there is large variation. Young people under the age of 20 years make up around a quarter of homicide victims [4]: 95,000 children were killed in 2012, a rate of eight per 100,000. Rates are particularly high in Latin America (12 per 100,000) and east and central Africa (10 per 100,000). The highest estimate is for El Salvador, at 27 per 100,000 [3]. In some countries, such as Venezuela, improvements in child health have been vitiated by child homicides [3].

We should view global estimates with caution as data are often incomplete, analysis requires assumptions, and misclassification is particularly likely with regard to homicide of young infants [3]. In general, homicides are likely to be underreported, particularly for neonates in countries that do not have complete coverage of birth registration and where deliveries take place outside institutions. Categorisation is always going to be elusive, but Abrahams and colleagues used the best methods they could, with an emphasis on conservative estimates. The researchers excluded cases for which no information was available and deaths ascribed to sudden infant death syndrome (of which up to 10% might represent homicide) [5], which could have produced an underestimate of the burden of child homicide. Conversely, they classified all cases of abandonment and subsequent death as homicide, which might have produced an overestimate.

The most common antecedent to death was abandonment of young infants, but there was little information on cause of death beyond this. Concealment of pregnancy is relatively common worldwide [6], and other studies have shown that suffocation and drowning are frequent methods of infanticide [7]. Causal inference is difficult if the child’s body is found in a partially decomposed state, and misclassification of stillbirths is possible. An assessment of abandoned fetuses and newborn infants in South Africa, by du Toit-Prinsloo and co-workers, found that 35% were decomposed. Amongst infants of greater than 26 weeks gestation, 28% (*n* = 31) were thought to have been born alive, but differentiation was not possible in 31% [8].

Abrahams and colleagues found no difference in child homicide rates by sex, although there was a decreased likelihood of male deaths in rural settings compared to urban settings. They rightly draw comparisons with south Asia and China, where both feticide and infanticide of girls have been a serious concern [9,10], but we should be cautious, given the modest size of Abrahams and colleagues’ study. In a related paper, Mathews and co-workers describe the epidemiology of child homicide in South Africa [11]. The pattern of homicide is similar for boys and girls, but changes with age. The homicide rate amongst males aged 15–17 years was five times that for females of the same age.

Abrahams and colleagues found—as have others—that mothers were the perpetrators in two-thirds of cases (maternal filicide). Studies from high-income countries suggest that the characteristics of mothers implicated in infanticide at birth and homicides of older infants are different. Women who commit neonaticide—the bulk of deaths—are more often young, unemployed or in school, and unmarried. Women implicated in the homicide of older infants tend to be older, and the homicide often occurs within a cycle of abuse [7]. The association of infanticide with maternal mental health is complex, and some studies support a link, while others do not [6]. Some women who commit infanticide are living with mental illness, including frank psychosis, but most infanticide does not seem to be associated with overt maternal mental illness [7].

What can the health community do? There are two general approaches: child protection and law enforcement, and primary prevention. Protecting vulnerable children is a priority, with an emphasis on supporting under-resourced and sometimes nonexistent child protection services, as is convicting perpetrators. In many cases, primary prevention of homicide through work with parents and families may be the best approach. Referring to deaths caused by parents, Resnick suggested a classification that included altruistic motives (to relieve suffering), acute psychosis, unwanted pregnancy, fatal consequences of child maltreatment, and revenge against another person, often a spouse [12]. Each of these categories has implications for the way we think about potential public health approaches. Some countries allow women to leave their infants anonymously in a safe place. The USA, for example, has introduced “safe havens” where infants can be abandoned legally. The effectiveness of such initiatives has yet to be determined, and it is not known whether mothers who might commit infanticide would call on them [13].

We agree with Abrahams and colleagues that more funds should go into maternity services, and also suggest that interventions need to be instigated before conception. As many births are unwanted, accessible and contextually appropriate family planning interventions are needed. Much work needs to be done with adolescent women to provide advice and support on sexual health, contraception, and childbirth. For women who present antenatally, a mental health assessment should be part of routine practice, with extra support for those in whom conditions are diagnosed or predicted [14]. Mortality data should be disaggregated and include homicide statistics, even if the numbers are small, so that we can move forward with a clearer picture of where interventions would yield the greatest benefit. We know a little, but not enough.
